# Implications of FBXW7 in Neurodevelopment and Neurodegeneration: Molecular Mechanisms and Therapeutic Potential

**DOI:** 10.3389/fncel.2021.736008

**Published:** 2021-08-25

**Authors:** Yu Yang, Xuan Zhou, Xinpeng Liu, Ruying Song, Yiming Gao, Shuai Wang

**Affiliations:** ^1^Shandong Collaborative Innovation Center for Diagnosis, Treatment and Behavioral Interventions of Mental Disorders, Institute of Mental Health, Jining Medical University, Jining, China; ^2^Shandong Key Laboratory of Behavioral Medicine, School of Mental Health, Jining Medical University, Jining, China; ^3^Research Center for Quality of Life and Applied Psychology, School of Humanities and Management, Guangdong Medical University, Dongguan, China

**Keywords:** FBXW7, E3 ubiquitin ligase, neurodevelopment, neurodegenerative disorders, therapeutic approach

## Abstract

The ubiquitin-proteasome system (UPS) mediated protein degradation is crucial to maintain quantitive and functional homeostasis of diverse proteins. Balanced cellular protein homeostasis controlled by UPS is fundamental to normal neurological functions while impairment of UPS can also lead to some neurodevelopmental and neurodegenerative disorders. Functioning as the substrate recognition component of the SCF-type E3 ubiquitin ligase, FBXW7 is essential to multiple aspects of cellular processes via targeting a wide range of substrates for proteasome-mediated degradation. Accumulated evidence shows that FBXW7 is fundamental to neurological functions and especially implicated in neurodevelopment and the nosogenesis of neurodegeneration. In this review, we describe general features of FBXW7 gene and proteins, and mainly present recent findings that highlight the vital roles and molecular mechanisms of FBXW7 in neurodevelopment such as neurogenesis, myelination and cerebral vasculogenesis and in the pathogenesis of some typical neurodegenerative disorders such as Alzheimer’s disease, Parkinson’s disease and Huntington’s disease. Additionally, we also provide a prospect on focusing FBXW7 as a potential therapeutic target to rescue neurodevelopmental and neurodegenerative impairment.

## Introduction

Proteolysis plays critical roles in diverse cellular processes including cell division, growth, differentiation and senescence. The ubiquitin–proteasome system (UPS) spatially and temporally controls a vast majority of protein degradation (Pohl and Dikic, 2019). Proteasomal degradation pathway is regulated by targeted ubiquitylation which undergoes a multi-step process participated by three key enzymes: an ubiquitin-activating enzyme (E1), an ubiquitin-conjugating enzyme (E2) and an ubiquitin ligase (E3). Ubiquitin, an evolutionally conserved protein of 76 amino acids is firstly activated by E1 with ATP causing a thioester bond between E1 and ubiquitin. Then, the ubiquitin is transferred to E2, and sequentially covalently binds to the ε-amino group of specific lysine residue on target protein by E3 (Li et al., 2018). E3 ligase determines the specificity of the ubiquitylation to target protein, and estimated over 600 E3 ligase genes are identified in human genome (Li et al., 2008).

UPS play vital roles in maintaining neurological functions while dysregulation and dysfunction of UPS components are involved in several neurodevelopmental and neurodegenerative disorders. For example, mutations, deletions, and duplications of *UBE3A* E3 ligase gene can lead to three human neurodevelopmental disorders: Prader-Willi syndrome (PWS), Angelman syndrome (AS) and Dup15q syndrome (LaSalle et al., 2015). E3 ligase HERC1 (regulator of chromosome condensation 1-like domain-containing protein 1) deficiency presents with delayed and abnormal brain development in mouse model (Bachiller et al., 2015), and patients with *HERC1* mutations present with thicker corpus callosum, seizures, intellectual disability, and other autism-resembling clinical symptoms (Ortega-Recalde et al., 2015; Aggarwal et al., 2016; Utine et al., 2017). Meanwhile, neurodegenerative diseases which can be commonly featured with aberrant aggregation of neurotoxic proteins in the central nervous system (CNS) have also been widely recognized to be associated with impairment of ubiquitin-proteasome system (McKinnon and Tabrizi, 2014; Harrigan et al., 2018). These evidence highlight the possibility that UPS components especially specific E3 ligases may be valid therapeutic targets for the treatment of neurodevelopmental and neurodegenerative disorders.

**FIGURE 1:**
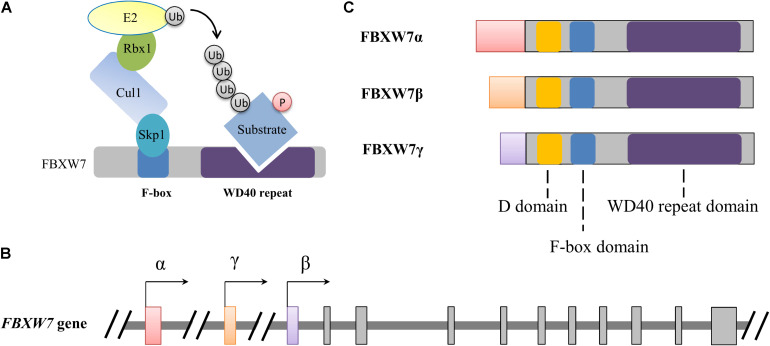
Schematic illustration of the SCF^FBXW7^ E3 ubiquitin ligase complex **(A)** and FBXW7 gene **(B)** and proteins **(C)**. **(A)** Functional model of FBXW7 involved in SCF complex-mediated substrate ubiquitylation. Ubiquitin and phosphorylation is indicated with Ub and P with a circle, respectively. **(B,C)** Alternative transcriptional initiations from different promoters generate three distinct FBXW7 transcripts and corresponding proteins with conserved D, F-box and WD40 repeat domains. All three FBXW7 transcripts (or isoforms) share 10 common exons which were shown with gray rectangles.

Broadly, E3 ligases are typically grouped into three major classes: the homologous to the E6AP carboxyl terminus (HECT) domain containing E3s, the really interesting new gene (RING) domain containing E3s and RING-between-RING (RBR) family E3s (Berndsen and Wolberger, 2014). Cullin-RING ubiquitin ligases (CRLs) belong to RING type E3 ligases. CRL1, also termed as the Skp1-Cullin 1-F-box protein (SCF) complex, is the most studied member among CRLs. The SCF complex consists of the S-phase kinase-associated protein 1 (Skp1), ring-box 1 (Rbx1), and Cullin 1 (Cul1), as well as a variable F-box protein which is responsible for substrate recognition (Figure 1). F-box and WD repeat domain containing 7 (FBXW7), also known as FBW7, AGO, hCDC4, and SEL-10, is one of the F-box proteins composing SCF type of E3 ubiquitin ligases (Shimizu et al., 2018). FBXW7 has been well studied for its crucial suppressive roles in tumorigenesis (Yumimoto and Nakayama, 2020). Meanwhile, mounting studies have focused on its functional roles in nervous system, especially suggesting that FBXW7 is crucial to neurodevelopment and neurodegeneration. In this review, we present evidence on the functional implications of FBXW7 in crucial neurodevelopmental processes and in the pathogenesis of some neurodegenerative disorders and also discuss the critical issues for drug development by targeting FBXW7, aiming to propose the therapeutic potential of targeting FBXW7 to ameliorate neurodevelopmental and neurodegenerative impairments.

## The FBXW7 Gene and Proteins

The *FBXW7* gene, spanning 216,330 bp of genomic DNA is located on chromosome 4q31.3. Alternative transcriptional initiations from different promoters and selective splicing generate three distinct *FBXW7* transcripts (Spruck et al., 2002). These transcripts share 10 common exons which are responsible for the encoding of conserved C-terminal region of FBXW7 proteins (FBXW7α, FBXW7β, and FBXW7γ). Alternative transcription determines the distinguished distribution of the three subtypes in tissues: FBXW7α widely expresses in almost all tissues, FBXW7β only exists in the brain and testes, and FBXW7γ is mainly detected in the heart and skeletal muscle (Jin et al., 2004; Matsumoto et al., 2006). All these isoforms share three crucial functional domains: a D domain mediating FBXW7 dimerization, a F-box domain interacting with the SKP1-CUL1 complex, and a tryptophan-aspartic acid 40 (WD40)-repeat domain which is responsible for substrate recognition and binding (Figure 1). The distinct N-terminal sequence of each isoform determines subcellular localization, with FBXW7α primarily in the nucleoplasm, FBXW7β in cytoplasm (or more precisely on the endoplasmic reticulum membrane), and FBXW7γ in the nucleolus (Welcker et al., 2004; Matsumoto et al., 2011b). As the result of wide distribution and dominant level of FBXW7α, plenty of known functions of FBXW7 are attributable to the α isoform while the β and γ subtypes may also irreplaceably contribute distinct roles in some specific physiological processes (Matsumoto et al., 2011b; Welcker et al., 2011; Xu et al., 2020).

## Regulation of FBXW7 Expression

The expression of FBXW7 is regulated at transcriptional, translational and post-translational levels. The CCAAT/enhancer binding protein-δ (C/EBPδ), an inflammatory response transcription factor, targets the *FBXW7*α promoter and directly inhibits *FBXW7*α transcription (Balamurugan et al., 2010). A functional p53-binding site was also identified in the 1st exon of β transcript of *FBXW7* gene, and p53 was confirmed to directly promote *FBXW7*β transcription (Kimura et al., 2003). Similarly, bHLH transcription factor 5 (HES5), a member of the HES family, was found to bind to *FBXW7*β promoter and suppress the *FBXW7*β transcription, although the specific binding sites have not been identified (Sancho et al., 2013; Chen et al., 2021). Additionally, the CpG sequences in the promoter region of *FBXW7*β were also proven to be methylated, leading to *FBXW7*β transcriptional suppression (Gu et al., 2008).

Multiple non-coding microRNAs (miRNAs) can modulate FBXW7 translation via interacting with the 3′ untranslated region of the mRNA. miR-24 (Zhao et al., 2016), miR-25 (Xiang et al., 2015; Hua et al., 2017; Peng et al., 2019), miR-27 (Wu et al., 2015; Liu Z. et al., 2018), miR-32 (Hua et al., 2016; Xia et al., 2017), miR-92 (Yang et al., 2015; Zhou et al., 2015), miR-129-5p (Hasler et al., 2012), miR-155-3p (Cao et al., 2016; Tang et al., 2016), miR-182 (Jeon et al., 2013; Li et al., 2014; Chang et al., 2018), miR-195-5p (Wang et al., 2019), miR-223 (Xu et al., 2010; Mansour et al., 2013), miR-367 (Xiao et al., 2017; Xu et al., 2017), miR-424 (miR-322 in mice) (Chen et al., 2019), miR-503 (Li et al., 2014), miR-544a (Liu X. et al., 2018), miR-586 (Zhang et al., 2016), and miR-1290 (Zhang et al., 2021) were shown to reduce FBXW7 protein level in different cancer cells. Adversely, some long non-coding RNAs (lncRNAs), such as MIF, TINCR, CASC2, MALAT1, and MT1JP can block the inhibition of FBXW7 expression acting as miRNA “sponges” (Cao et al., 2016; Wang et al., 2017; Liu X. et al., 2018; Zhang et al., 2018). Moreover, FBXW7 translation is also regulated by mRNA modification. N6-methyladenosine (m^6^A) modification mediated by METTL3 (methyltransferase-like 3) was proven to enhance FBXW7 translation (Wu et al., 2021).

Post-translational regulation of FBXW7 includes ubiquitination, phosphorylation and dimerization of the proteins. First, ubiquitination and deubiquitination of FBXW7 regulate its proteasomal degradation. COP9 signalosome complex subunit 6 promotes FBXW7 autoubiquitination and proteasome-mediated degradation. Ubiquitin specific peptidase 28 (USP28), a deubiquitinating enzyme can also repress autocatalytic ubiquitination and degradation of FBXW7 (Diefenbacher et al., 2014; Schulein-Volk et al., 2014). Moreover, the stability and function of FBXW7 are regulated by multiple kinases. For example, extracellular signal–regulated kinase (ERK) and Polo-like kinase 1 and 2 (PLK1 and PLK2) directly interact and thereby mediate phosphorylation of FBXW7, resulting in its ubiquitination and proteasomal degradation (Cizmecioglu et al., 2012; Ji et al., 2015; Xiao et al., 2016). In contrast, phosphorylation of FBXW7α by phosphoinositide 3-kinase (PI3K) and serumand glucocorticoid-regulated kinase 1 (SGK1) were demonstrated to inhibit its autocatalytic ubiquitin transfer and stabilization (Mo et al., 2011; Schulein et al., 2011). Besides, the dimerization mediated by the D domain in all three FBXW7 isoforms also affects the stability of FBXW7. Peptidylprolyl cis/trans isomerase NIMA-interacting 1 (Pin1) has been demonstrated to destabilize FBXW7 by repressing dimerization and thereby promoting FBXW7 autoubiquitination (Min et al., 2012). Similarly, FBXW7 monomers were found to be stable as dimerization could destabilize the protein because of accelerated autoubiquitylation (Welcker et al., 2013).

## FBXW7 in Neurodevelopment

Mounting studies indicate the homeostasis of FBXW7 is crucial for neurodevelopment. In this section, we will present evidence of functional involvement of FBXW7 in neurodevelopment and review the underlying mechanisms of FBXW7 in the processes of neurogenesis, myelination, and cerebral vasculogenesis. Additionally, potential therapeutic effect on neurodevelopmental disorders treatment via targeting FBXW7 or its substrates is also discussed.

### Structural and Functional Abnormalities in FBXW7-Deficient Mouse Brain

Mice with specific deletion of exon 5 of *Fbxw7* in brain die in a short while after birth with substantial changes and morphological abnormities in brain structure. Neurogenesis was found to be defective while astrogenesis was enhanced, leading to the tendentious differentiation toward astrocytes in these conditional Fbxw7-deficient brain. These newborn Fbxw7-deficient mice also show defective suckling behavior which may be associated with the hypoplasia of the brain stem although the underlying cause of the defective behavior remains to be explored (Matsumoto et al., 2011a). Similarly, it was also reported that conditional inactivation of *Fbxw7* in the nervous system resulted in severely defective stem cell differentiation and anabatic progenitor cell death. Neurospheres from Fbxw7 deficient embryos were generally smaller in size, and significantly lower in number (Hoeck et al., 2010). Moreover, conditional *Fbxw7*-knockout in the cerebellar anlage of mouse leaded to reduced Purkinje cell number, decreased cerebellar size and defects in axonal arborization. Fbxw7-deficient cerebella presented with reduced vermis size and aberrant migration of progenitor cells (Jandke et al., 2011). Besides, primary cultures of neurons prepared from the mice only lacking β isoform of Fbxw7 were more vulnerable to oxidative stress although rare morphological abnormalities exhibit in brain development (Matsumoto et al., 2011b).

### FBXW7 and Neurogenesis

Initial expansion of the progenitor cell by symmetrical division and subsequent generation of differentiated cells such as neurons, astrocytes, and oligodendrocytes through asymmetrical division are crucial to brain development (de la Pompa et al., 1997; Lutolf et al., 2002). The functional implication of FBXW7 in brain development is crucially mediated by Notch and c-Jun, both of which are substrates of FBXW7. The Notch signaling pathway acts via a process of lateral inhibition to play a fundamental role in neuronal and glial differentiation. Dll1, a ligand of Notch can trigger Notch signaling and suppress the expression of the proneural genes via inducing Hes1 expression and thereby block neuronal differentiation (Kageyama et al., 2009). Notch signaling inactivation promotes premature neurogenesis, leading to exhaustion of the progenitor pool and decreased number of mature neurons (de la Pompa et al., 1997; Lutolf et al., 2002). FBXW7 regulates Notch protein stability in this process, thereby controlling the maintenance and differentiation orientation of neural stem cells. Notch accumulation caused by FBXW7 deficiency results in aberrant activation of Notch target genes, resulting to excessive proliferation of neural stem cells and aberrant differentiation toward the astrocytes (Matsumoto et al., 2011a). c-Jun is another important regulator of neuronal viability. Restraining of c-Jun activation prominently rescued the cellularity defect caused by *Fbxw7* deletion in the mantle layer of the midbrain tectum. Likewise, compared to *Fbxw7* single mutant, *Fbxw7*/*Jun* mutant cells exhibited substantially elevated neurosphere formation *in vitro*, accompanied by a considerable reduction of apoptotic cells in neurospheres, indicating that c-Jun–mediated cell death is functionally implicated in defective neurosphere formation under Fbxw7 deficiency background (Hoeck et al., 2010). Besides, it was also reported that deletion of c-Jun or specific abrogation of c-Jun N-terminal phosphorylation could rescue Purkinje cell numbers and arborization in the *Fbxw7* knockout background indicating phosphorylated c-Jun is an important substrate of Fbxw7 in neurogenesis during cerebellar development (Jandke et al., 2011). WD repeat domain 62 (WDR62) is crucial to promoting c-Jun N-terminal kinase signaling in the control of neurogenesis (Wasserman et al., 2010; Cohen-Katsenelson et al., 2011). It was also reported that FBXW7 controls self-renewal and differentiation of neural progenitor cells (NPCs) during brain development by regulating WDR62 degradation (Xu et al., 2018).

### FBXW7 and Myelination

Myelin, a specialized proteolipid-rich membrane surrounding neuronal axons, is crucial for axons protection and insulation. Myelination is a sophisticated solution for efficient conduction velocity of potential actions along axons. Myelin is formed by glial cells which are oligodendrocytes and Schwann cells in the central nervous system and peripheral nervous system, respectively.

During myelin development in central nervous system, specified oligodendrocyte precursor cells (OPCs) migrate to target axons before they begin to differentiate into premyelinating oligodendrocytes which wrap axons and synthesize multiple myelin proteins and lipids comprising the myelin sheath. Consequently, oligodendrocytes can form multitudinous myelin sheaths with substantial variability in lengths and thicknesses (Kessaris et al., 2006; Hughes et al., 2013; Hughes and Appel, 2019). Notch signaling mediated by Notch protein and its receptors plays a vital role in balancing development of neurons and glia. Dysfunction of Notch pathway in vertebrate embryos generally leads to reduced neural precursors, excessive early born neurons and a deficit of glial cells, oligodendrocytes included (Chitnis et al., 1995; de la Pompa et al., 1997; Park and Appel, 2003). It was reported that *fbxw7* mutation leaded to excessive differentiation of neural precursors toward oligodendrocyte progenitor cells in zebrafish embryos nearly identical to that of the mutant with constitutive activation of Notch (Park and Appel, 2003). Hyperactive Notch signaling was found in *fbxw7* mutant embryos while pharmacological inhibition of Notch proteins under *fbxw7* mutant background inhibited formation of excess oligodendrocyte progenitors indicating that Notch signaling are functional target of Fbxw7 in the process of oligodendrocyte specification (Snyder et al., 2012). Similarly, mTOR (mammalian target of rapamycin) is another target of FBXW7 in regulating oligodendrocyte differentiation. mTOR signaling is also promoted in oligodendrocyte lineage cells of *fbxw7* mutant zebrafish larvae. Both genetic and pharmacological inhibition of mTOR signaling are beneficial to rescue aberrant profiles of myelin genes caused by dysfunction of Fbxw7, indicating that mTOR is a functional target of Fbxw7 in oligodendrocytes (Kearns et al., 2015).

In peripheral nervous system, myelin is fundamentally developed from Schwann cells (SCs). Neural crest precursor cells initially proliferate and differentiate into SC precursors which then differentiate into immature SCs. The maturation of SC comes up around birth via the process of radial sorting, during which the cytoplasmic components of individual SCs extend into bundles of axons, progressively separates them into smaller bundles, and finally surrounds a single larger diameter axon (Martin and Webster, 1973; Webster et al., 1973; Chernousov et al., 2008). Meanwhile non-myelinating SCs will form into Remak bundles by ensheathing multiple small diameter axons (Jessen and Mirsky, 2005). Conditional knockout of *Fbxw7* specifically in SC precursors at approximately embryonic day (E) 12.5 results in thicker myelin sheaths and a higher proportion of myelinated axons compared to control nerves. More intriguingly, *Fbxw7* mutant SCs sometimes appear to myelinate multiple axons in a fashion reminiscent of oligodendrocytes. It is identified that Fbxw7 regulates mTOR to control SC number, myelination, and Remak bundle organization during myelination peripheral nervous system. The activation of c-Jun is also found in Fbxw7 mutant SCs while the potential role of c-Jun in regulating SCs needs further demonstration (Harty et al., 2019). These evidences indicate that FBXW7 functionally regulate plasticity of SCs during myelination and may be a beneficial target for myelin repair.

### FBXW7 and Cerebral Vasculogenesis

FBXW7 also control vasculogenesis in brain. Apart from metabolic functions of ensuring adequate supply of oxygen and nutrients to maintain homeostasis of neuronal networks, vessels have also been considered to serve as niches and scaffolds for neuronal migration and expansion during brain development and neurogenesis (Attwell et al., 2010; Segarra et al., 2015, 2018). *Fbxw7*-null mice (*Fbxw7*^–/–^ with disruptions of all three isoforms) die *in utero* at embryonic day around 10.5 as a result of impaired vascular development in the brain and yolk sac. *Fbxw7*^–/–^embryos shows defects of vessels along the entire length of their neural tubes, indicating FBXW7 is potentially fundamental for brain function by regulating construction of neurovascular architecture during development (Tetzlaff et al., 2004; Tsunematsu et al., 2004). However, the mechanism remains elusive at present.

### FBXW7 and Neurodevelopmental Disorders

It is undoubted that FBXW7 and its substrates function in different vital neurodevelopmental processes, but the potential roles of FBXW7 in the pathogenesis of neurodevelopmental disorders still remain to be investigated. There is no *FBXW7* mutation identified to be associated with any neurodevelopmental disorders at present, however, functional implications or mutations of its substrate have been widely reported in relevant diseases. For example, c-Jun was aberrantly increased in an autism mouse model (*Engrailed-2* knockout) (Tripathi et al., 2009), and c-Jun activation was possibly involved in the autism via inducing disordered inflammatory response in the brain (Shimoyama et al., 2019; Bjorklund et al., 2020). Also, some neurodevelopmental disorders such as focal cortical dysplasias, tuberous sclerosis complex and syndromic autism spectrum disorder (ASD) are thought to arise due to the effects of mTOR mutations during fetal development (Sato, 2016; Iffland and Crino, 2017; Salussolia et al., 2019). Similarly, mutations in Notch 3 can also lead to cerebral autosomal dominant arteriopathy with subcortical infarcts and leukoencephalopathy (Mizuno et al., 2020). Considering functional implications of FBXW7 in neurodevelopment, it may be beneficial to explore effective methods via targeting FBXW7 or its substrate for treatment of neurodevelopmental disorders. For instance, everolimus, an inhibitor of mTOR which is used for treatment of tumor manifestations in patients with tuberous sclerosis complex also provide impressive therapeutic effect on improving neuropsychiatric symptoms (Kilincaslan et al., 2017). Rapamycin, another mTOR inhibitor was also reported to prevent the pathological and behavioral deficits in ASD (Tsai et al., 2012). These emerging evidence suggests mTOR inhibitors could be a potential pharmacotherapy for ASD (Sato, 2016).

## FBXW7 in Neurodegeneration

Despite of dysgenopathy in nervous system, anabatic neurodegeneration is another prominent characteristic caused by FBXW7 dysfunction. A large body of evidence indicates FBXW7 may be implicated in the pathogenesis of some typical neurodegenerative diseases. Potential underlying pathways of FBXW7 involved in Alzheimer’s disease, Parkinson’s disease and Huntington’s disease are discussed in this part.

### Alzheimer’s Disease

Alzheimer’s disease (AD), the most common form of dementia especially in the old, affects more than 50 million people worldwide. AD patients are typically characterized with amyloid plaque and neurofibrillary tangles in the brain, both of which are regarded as two hallmarks of this disease (Drew, 2018). At present, dysregulation or dysfunction of FBXW7 has not been reported in AD patients or animal models, but some evidence may support the issue that FBXW7 is involved in the pathogenesis of AD.

Firstly, FBXW7 potentially regulates amyloid-β (Aβ) generation. Overexpression of FBXW7 in HEK293 cells could alter APP metabolism and lead to an increase in the production of Aβ (Li et al., 2002). However, the effect and mechanism of FBXW7 on Aβ production remains elusive so far. It has been widely recognized that the generation of Aβ, especially Aβ_42_, which aggregates into bioactive conformational species, likely initiates the toxicity in AD (O’Brien and Wong, 2011; Potter et al., 2013; Szaruga et al., 2015; Selkoe and Hardy, 2016; Makin, 2018). Aβ is generated from APP, successively processed by β-secretase and γ-secretase complex. BACE1 (β-site cleaving enzyme 1), a membrane-located aspartyl peptidase, acts as the dominated β-secretase which provides the first cleavage of APP at β-site (Zhang et al., 2021). Hypoxia-inducible factor 1 subunit α (HIF-1α), a factor induced by hypoxia, was reported to directly regulate *BACE1* transcription and is contributing to BACE1 upregulation in response to hypoxia in the pathogenesis of AD (Sun et al., 2006; Zhang et al., 2007). It indicates that FBXW7 may be implicated in Aβ generation by regulating BACE1 level in a HIF-1α dependent pathway. Moreover, Li et al. (2002) also showed that FBXW7 interacted with Presenilin 1 (PS1), a crucial component of γ-secretase, revealing FBXW7 may alter γ-secretase activity by binding to PS1 protein, thus promoting processing of APP and Aβ generation.

Furthermore, FBXW7 may regulate neuronal apoptosis which seems to be inordinate in AD brain (Obulesu and Lakshmi, 2014; Fricker et al., 2018). FBXW7 is known to be implicated in neuronal apoptosis. For example, FBXW7 can mitigate neuronal apoptosis by mediating c-Jun proteolysis in response to glutamate-induced excitotoxicity (Ko et al., 2019). c-Jun is known as a substrate of SCF^FBXW7^ and plays a crucial role in accelerating cell apoptosis (Bossy-Wetzel et al., 1997). Consistently, FBXW7 has also been confirmed to bind parkin in neurons and to collaborate with parkin to ubiquitylate and destabilize the target cyclin E1 (Staropoli et al., 2003). Excessive cyclin E1 accumulation in neurons can lead to neuronal apoptosis (Padmanabhan et al., 1999), especially under conditions of excitotoxicity, suggesting a neuroprotective role for FBXW7. Besides, Ko et al. (2020) demonstrated that Fbxw7 was cleaved by activated calpain in the ipsilateral cortex in the rat model with middle cerebral artery occlusion. Negative regulation of Fbxw7 by calpain leaded to neuronal cell death while the preservation of Fbxw7 by the inhibition of calpain or other strategies may provide a novel protective mechanism against aberrant cell apoptosis in response to excitotoxicity (Ko et al., 2020). Similarly, Fbxw7 level was significantly reduced in mice spinal cord tissues in response to spinal cord injury while enhanced Fbxw7 expression can effectively moderate the progression of spinal cord injury by repressing microglial inflammation and neuronal death (Chen et al., 2020). Additionally, FBXW7 possibly mitigate neuronal apoptosis via mediating proteasome-dependent degradation of regulator of calcineurin 1 (RCAN1). RCAN1, a crucial endogenous regulator of calcineurin (Wang S. et al., 2020), is highly expressed in human brain and is particularly aberrantly elevated in the brains of AD patients (Ermak et al., 2001; Harris et al., 2007). Several lines of evidence suggest that RCAN1 functions in neuronal apoptosis (Lee et al., 2007; Sun et al., 2011; Ermak et al., 2012; Wu and Song, 2013) and the degradation of RCAN1 proteins is mediated by both UPS and the chaperone-mediated autophagy pathways (Liu et al., 2009). RCAN1 is a specific target of FBXW7 in the process of ubiquitin-proteasome-mediated degradation (Lee et al., 2012; Hong et al., 2015). It suggests that FBXW7 may be responsible for dysregulation of RCAN1 in AD but the mechanism needs to be further investigated. However, the functional role of FBXW7 in neuronal apoptosis remains controversial. For instance, FBXW7β was also reported to promote neuronal apoptosis via mediating ubiquitylation-dependent proteolysis of Mcl-1. Mcl-1, a specific SCF^Fbxw7^ target in neurons, functions as a mitochondrial prosurvival factor in neuronal apoptosis (Ekholm-Reed et al., 2013). It suggests that functional implication of FBXW7 in neuronal apoptosis potentially depends on its subcellular localization and specific target affinity.

In addition, FBXW7 may also be involved in AD by modulating cell senescence. It is known that aging is the dominated risk factor for AD. Cell senescence is irreversible programed process which determines the aging process of the body. Telomere shortening plays a crucial role in cell senescence therefore telomere dysfunction is always associated with aging-related diseases (Blackburn et al., 2015; Tian et al., 2019). A recent work reported that FBXW7 mediated cell senescence through telomere uncapping. FBXW7 interacts with telomere protection protein 1 (TPP1), promotes TPP1 multisite ubiquitylation and degradation, and thereby triggers telomere uncapping and DNA damage response (Wang L. et al., 2020). However, the potential role of FBXW7 in neuronal senescence remains to be confirmed.

### Parkinson Disease

Parkinson’s disease (PD) is another most common neurodegenerative disorders, affecting over 1% of the population older than 60 years of age. PD is characterized by progressive degeneration of nigrostriatal dopaminergic (DA) neurons in the midbrain and clinically diagnosed as motor abnormalities including bradykinesia, resting tremor, and cogwheel rigidity (Duvoisin, 1992; Abou-Sleiman et al., 2006). Mutations in the *PARK2* gene are involved in a small portion of the cases which is known as autosomal recessive juvenile parkinsonism (ARJP) (Saito et al., 2000). It has been reported that FBXW7β levels are elevated in the cortexes of PD patients with a biallelic *PARK2* mutation. Parkin, the product of *PARK2* gene, is responsible for polyubiquitylating FBXW7β and targeting it for proteasomal degradation. Parkin deficiency-mediated FBXW7β elevation in some PD cases accelerates Mcl-1 degradation, subsequently leading to aberrant neuronal apoptosis (Ekholm-Reed et al., 2013). Intriguingly, it was also reported that FBXW7β protein level did not change in postmortem sporadic PD brains but FBXW7β was highly oxidized with excessive carbonyl formation. Similarly in the 6-hyroxydopamine (6-OHDA) induced PD mouse model, both of the total and oxidation level of FBXW7β decreased in the substantia nigra compacta. 6-OHDA enhanced the binding of FBXW7β with Hsc70, another fundamental regulator of chaperone-mediated autophagy (CMA), enabling the delivery of FBXW7β to LAMP2A and accelerating FBXW7β degradation mediated by CMA. However, the functional implication of oxidation-mediated FBXW7β reduction in the pathogenesis of PD deserves further investigation (Wang et al., 2018).

### Huntington’s Disease

Huntington’s disease (HD) is an inherited autosomal dominant neurodegenerative disorder caused by accumulated mutant Huntingtin (Htt) protein with a poly-glutamine expansion (encoded by CAG trinucleotide repeat) (Ross and Tabrizi, 2011). Mutant Htt upregulates CK2α kinase and FBXW7, which phosphorylates and ubiquitylates heat shock transcription factor 1 (HSF1), respectively, thus promoting its proteasome-mediated degradation. Consistently, HSF1 was downregulated in striatum and cortex from patients with HD, causally leading to neuronal dysfunction. It indicates that blocking FBXW7-mediated HSF1 degradation may effectively ameliorate defects in neuronal function and promote survival in HD (Gomez-Pastor et al., 2017). Moreover, FBXW7 is also implicated in HD by targeting p53 for degradation. A potential causal role of impaired mitochondrial fission caused by dysfunction of dynamin-related protein 1 (Drp1) in neuronal damage of HD has widely been suggested (Song et al., 2011; Shirendeb et al., 2012). p53, a stress sensor involved in HD pathogenesis, interacts with DRP1 to promote DRP1-induced mitochondrial and neuronal damage (Guo et al., 2013). p53 can be phosphorylated by GSK3 and ATM at serine 33, then ubiquitylated by SCF^FBXW7^ and degraded in the proteasomal pathway (Galindo-Moreno et al., 2019; Cui et al., 2020). This suggests that targeting FBXW7 for inhibiting p53 may prevent the progression of HD by suppressing DRP1-dependent excessive mitochondrial fission and neuronal damage.

## Challenges and Prospects on Drug Development by Targeting FBXW7

Considering the crucial roles of FBXW7 in neurodevelopment and neurodegeneration, FBXW7 may be a potential therapeutic target for neurodevelopmental and neurodegenerative disorders treatment. However, drug development by targeting FBXW7 is also faced with several challenges which may result from the following reasons. Firstly, FBXW7 functions in multiple physiological processes by targeting a variety of substrates therefore potential side effects of FBXW7 modulation should be considered. For example, FBXW7 is also regarded as a tumor suppressor and inactivation of FBXW7 can increase resistance to anti-tubulin drugs and promote tumorigenesis (Yumimoto and Nakayama, 2020). Moreover, a broad spectrum of the tissue-specific regulatory mechanisms and substrate selectivity of FBXW7 further increase the requirement for precise drug development. Besides, the functional heterogeneity of FBXW7 isoforms remains elusive although it would be more precise to target specific isoform of FBXW7 for amelioration of neurodevelopmental and neurodegenerative impairments.

**FIGURE 2:**
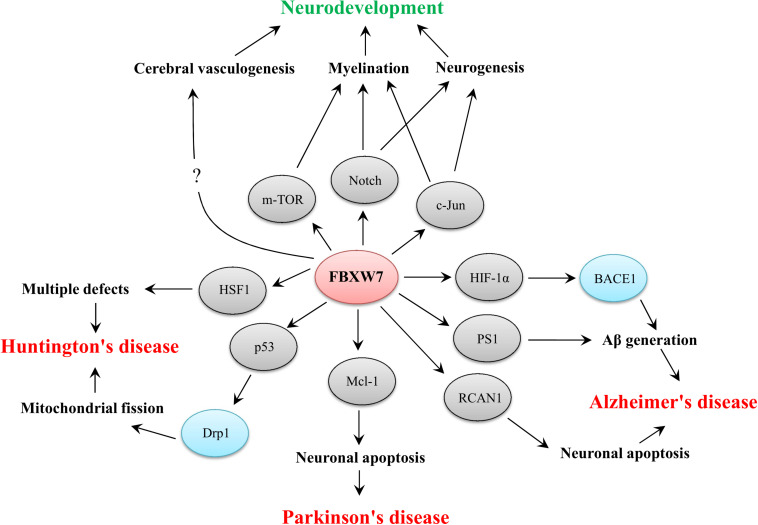
Functional implications of FBXW7 in neurodevelopment and three typical neurodegenerative diseases. The substrates of FBXW7 and other downstream factors are presented by gray or blue ellipses, respectively.

However, instead of directly modulating FBXW7 level or activity, interventions on interactions between FBXW7 and its targets may provide a more feasible therapeutic strategy. For example, some specific oligopeptides which are designed based on the degron motif within the substrate are confirmed to effectively inhibit substrates degradation via competing binding with FBXW7 (Yalla et al., 2018; Wang L. et al., 2020). As a consequence, the development of chemical inhibitors or oligopeptides targeting FBXW7 by inhibiting SCF^FBXW7^ mediated substrate degradation should shed light on the therapeutic potential of targeting FBXW7-mediated degradation for the treatment of neurodevelopmental and neurodegenerative disorders. For example, blocking the FBXW7 mediated HSF1 and Mcl-1 degradation may effectively ameliorate defects in neuronal function in HD and PD, respectively, even though effective acceleration of HIF-1α, PS-1 and RCAN1 by targeting FBXW7-mediated degradation possibly provides curative effect on AD treatment (Figure 2). Meanwhile, considering the therapeutic effect of mTOR inhibitors on ameliorating autism-like symptoms, appropriate induction of FBXW7-mediated degradation of mTOR may also be beneficial for autism treatment but the effect still remains to be demonstrated.

## Conclusion

Accumulating evidence has shown that FBXW7 functions in neurodevelopment and neurodegeneration. In summary, FBXW7 is not only implicated in neurodevelopment by regulating neurogenesis, myelin development and cerebral vasculogenesis but also involved in the pathogenesis of some neurodegenerative disorders, such as AD, PD, and HD (Figure 2). Thus, targeting FBXW7 or FBXW7-substrate interaction may offer the opportunities for drug development against neurodevelopmental and neurodegenerative impairments even though some challenges also deserve further consideration.

## Author Contributions

YY and SW wrote the manuscript and made the figures. XZ, XL, RS, and YG revised and approved the manuscript. All authors contributed to the article and approved the submitted version.

## Conflict of Interest

The authors declare that the research was conducted in the absence of any commercial or financial relationships that could be construed as a potential conflict of interest.

## Publisher’s Note

All claims expressed in this article are solely those of the authors and do not necessarily represent those of their affiliated organizations, or those of the publisher, the editors and the reviewers. Any product that may be evaluated in this article, or claim that may be made by its manufacturer, is not guaranteed or endorsed by the publisher.
